# Within-session verbal learning slope is predictive of lifespan delayed recall, hippocampal volume, and memory training benefit, and is heritable

**DOI:** 10.1038/s41598-020-78225-1

**Published:** 2020-12-03

**Authors:** Kristine B. Walhovd, Anne Cecilie Sjøli Bråthen, Matthew S. Panizzon, Athanasia M. Mowinckel, Øystein Sørensen, Ann-Marie G. de Lange, Stine Kleppe Krogsrud, Asta Håberg, Carol E. Franz, William S. Kremen, Anders M. Fjell

**Affiliations:** 1 Center for Lifespan Changes in Brain and Cognition, Department of Psychology, University of Oslo, POB 1094, 0317 Oslo, Norway; 2 Division of Radiology and Nuclear Medicine, Oslo University Hospital, Rikshospitalet, Norway; 3 Department of Psychiatry and Center for Behavior Genetics of Aging, University of California, San Diego, USA; 4 Department of Neuroscience, Norwegian University of Science and Technology (NTNU), Trondheim, Norway; 5 Present Address: Department of Psychiatry, University of Oxford, Oxford, UK; 6 Present Address: Department of Psychology, University of Oslo, Oslo, Norway

**Keywords:** Cognitive ageing, Cognitive neuroscience, Learning and memory, Neuroscience, Biomarkers, Psychology, Human behaviour

## Abstract

Memory performance results from plasticity, the ability to change with experience. We show that benefit from practice over a few trials, *learning slope*, is predictive of long-term recall and hippocampal volume across a broad age range and a long period of time, relates to memory training benefit, and is heritable. First, in a healthy lifespan sample (n = 1825, age 4–93 years), comprising 3483 occasions of combined magnetic resonance imaging (MRI) scans and memory tests over a period of up to 11 years, learning slope across 5 trials was uniquely related to performance on a delayed free recall test, as well as hippocampal volume, independent from first trial memory or total memory performance across the five learning trials. Second, learning slope was predictive of benefit from memory training across ten weeks in an experimental subsample of adults (n = 155). Finally, in an independent sample of male twins (n = 1240, age 51–50 years), learning slope showed significant heritability. Within-session learning slope may be a useful marker beyond performance per se, being heritable and having unique predictive value for long-term memory function, hippocampal volume and training benefit across the human lifespan.

## Introduction

Memory function dependent on medial temporal lobe (MTL) structures is target of intensive research on normal as well as degenerative age changes in cognition^1–3^. Yet, surprisingly little attention has been paid to the individual differences and characteristics of the learning process underlying performance on memory tests. There are substantial individual differences in episodic memory performance as well as the hippocampal formation^4–7^. While a major portion of both can be accounted for by genetic factors^8,9^, episodic memory is inherently derived from experience, and how much one changes with experience will necessarily be critical to one´s memory performance. Both genetic and environmental factors will be at play in this. There is evidence for heritability of acquisition and retrieval of verbal information, and specific genetic influences on acquisition have been indicated^10^. However, little is known of heritability of verbal learning slope, or ability to benefit from practice. We know that practice may dramatically alter retrieval, as evidenced by effect on recall performance. Training studies show that recall performance can be more than doubled with strategy training^11,12^. However, there is huge individual variability in training responses^11^, and beyond a few proximate characteristics, we know little of what makes some individuals’ memory benefit more from practice than others, and to what extent this variability matters in the long run. This is surprising, as the human MTL, its connections, and MTL-dependent memory are vulnerable in aging^13–24^, and have unique plastic characteristics^16,25–27^. It is now pivotal that the predictive value across the lifespan and the heritability of MTL-based learning slope is investigated in a controlled setting.

### Unknown relations between learning slope within-session and across weeks, standard measures of delayed recall, and hippocampal volumes

Much research has focused on single measures of free recall^28^. These standard measures typically refer to how much can be remembered of a stimulus material, such as a verbal list, shortly after presentation, or after an interval of maximum 20–30 min (termed delayed recall)^29,30^. However, such metrics often derive from list learning tests that comprise several trials, enabling not only single measures of recall or aggregate measures of total learning, but also assessment of learning slope, i.e. cognitive plasticity. Cognitive plasticity can be defined in terms of the individual´s latent cognitive potential or capacity to acquire skills under specific contextual conditions^31^. In accordance with Baltes and Lindenberger^32^, the focus is on *intraindividual modifiability*, the range of performance or potential within an individual to change performance.

Various methods have been used to measure such interindividual modifiability of performance, involving repeated exposure or training with a range of different stimuli or tasks, targeting anything from sensory, motor, and attentional processes to higher order cognitive strategies, yielding multiple plasticity paradigms^31,33–36^. Typically, cognitive plasticity has been studied within a short time frame^31^. Learning slope across trials within one session is an example of short-term plasticity, and differences in learning curves with age have been characterized^37^. There are indications that learning slope may be more related to neuroanatomical features in relatively early neurodegenerative conditions, such as mild cognitive impairment (MCI), than in normal aging^38^. In a recent cross-sectional population-based study of healthy older adults, it was found that hippocampal volume positively predicted verbal learning rates (estimated by a latent growth model), but only in individuals with higher limbic white matter anisotropy^39^. It is unknown how learning slope relates to delayed recall and hippocampal volumes in a cognitively healthy lifespan population. Moreover, range of cognitive plasticity manifested is expected to vary on the basis of duration, intensity, or instructional procedures used^31^, and it is unknown how short-term cognitive plasticity, i.e. modifiability of performance across trials within one session, relates to cognitive plasticity as measured in another context, across weeks of cognitive training, here termed long-term plasticity. It may be expected that relatively short-term cognitive plasticity measured in terms of verbal learning slope across trials within one session, should relate positively to long-term plasticity measured in terms of improvement in learning verbal material across weeks or months. This is so because both short-term and long-term plasticity as measured here to some extent rely on the medial temporal lobe system underlying explicit, episodic memory^40^. However, there are important differences. While the within-session across-trial learning only involves repetition and testing of the same material, the training across weeks involves learning strategies for encoding and recalling, and testing of different materials. It has been shown that mnemonic training drives distributed changes^41^. For these reasons, one may expect a relationship between within-session learning slope and long-term memory training benefit, but such a relationship may not be strong, as additional factors, and additional brain areas, would be involved in long-term memory training.

### Unknown heritability of verbal learning slope

Given the established heritability of brain and cognition, one may expect that one´s potential for modifying performance is heritable too. As genetic influences work at multiple levels^42–44^, studies including measures of change over multiple opportunities to learn are critical to address this issue. A genetic component for neurocognitive plasticity has previously been indicated in training studies of candidate genes^35,45–48^. However, findings have in part been inconsistent^49^. There are limitations to the candidate gene approach, and training samples are typically not large enough for genome-wide association studies (GWAS). Valuable independent and complementary information on genetic influences on neurocognitive plasticity may be gained from twin data of episodic memory practice effects, i.e. learning slope. So far, the few twin studies that have investigated practice effects have centred on basic motor learning or conditioning. A seminal paper on acquisition of skill for rotary pursuit^34^ reported that heritability increased with number of trials. A small study of young adult twins^36^ used paired associative stimulation of motor cortex to elicit motor evoked potential in resting muscle. The derived heritability estimate for brain plasticity was 0.68, implicating that genetic factors may contribute significantly to inter-individual variability in plasticity paradigms^36^. One cannot, however, readily generalize from heritability of sensory or motor modifiability to modifiability of complex cognitive processes. There are to our knowledge no previous studies testing the heritability of neurocognitive plasticity or practice effects on hippocampal-based memory.

### Investigating whether learning slope is predictive of lifespan delayed recall, hippocampal volume, and memory training benefit, and is heritable

Here we quantify learning slope as the amount of benefit from practice over trials in a verbal recall task. We investigate how learning slope predicts delayed recall and hippocampal volume across the lifespan. We investigate whether learning slope is associated with long-term benefit measured experimentally in a memory training paradigm across ten weeks. We also investigate whether learning slope shows significant heritability and whether heritability of memory increases with learning. We utilize data from three different samples: (1) a *lifespan sample* followed longitudinally, to assess relations between single-session learning rate, and long-term hippocampal and memory characteristics, (2) an experimental subsample of the lifespan sample undergoing extended memory training across 10 weeks, i.e. a *training sample,* to assess the relations between single-session learning rate and long-term memory training benefit, and (3) a *twin sample*, of middle-aged men, to assess the heritability of learning rate. We hypothesize that rate of learning slope as assessed in a single session has unique predictive value for memory and hippocampal volumes across the lifespan, is positively related to benefit from long-term memory training, and is heritable.

## Results

### Analyses in the lifespan sample: Learning slope is positively related to longitudinal delayed recall and hippocampal volume through the lifespan

At all test occasions, a verbal learning test comprising a list of 16 items was given (see “Methods”). The list was read five times, and learning slope was quantified as performance after last learning trial subtracted from the performance after first learning trial (trial 5 minus trial 1, i.e. trial 1 is termed “offset”). Total learning was defined as the sum of items recalled across all learning trials (max 80), and delayed recall was quantified as number of items remembered after approximately 20 min (see “Methods”). The overall relationships in terms of correlations between variables of interest are shown in Supplementary Table 1. First, the relationships between learning slope, delayed recall and hippocampal volume were tested in the cognitively healthy *lifespan sample* (for a description of distribution of scans and tests across the decade of follow-up see Table 1). For all results where confidence intervals (CI) of effect sizes are reported, 95% CI is used.

**Table 1:** Lifespan sample descriptive characteristics across 3483 observations.

	Time point 1 (n = 1825, 1098 F)		Time point 2 (n = 888, 508 F)		Time point 3 (n = 423, 251 F)		Time point 4 (n = 174, 97 F)		Time point 5 (n = 116, 69 F)		Time point 6 (n = 57, 30 F)	
	M	Range	M	Range	M	Range	M	Range	M	Range	M	Range
Age	29.8	4.1–93.3	36.2	5.5–88.5	45.7	10.7–82.3	60.6	21.8–83.3	59.0	22.1–82.8	63.2	23.1–84.0
Interval	–	–	2.7	0.2–9.5	4.7	0.4–10.0	2.6	0.6–11.0	1.6	0.8–6.6	3.0	1.8–8.9
Hip. vol	8134	4494–11,607	8037	4447–10,920	7976	4400–11,417	7641	4375–10,747	7667	5601–10,801	7564	5584–10,735
Learn. trial 1	6.8	0–16	7.2	0–16	7.6	2–15	7.0	2–15	7.4	2–15	7.4	3–12
Learn. slope	5.7	− 5 to 14	6.1	− 4 to 14	6.3	− 1 to 14	7.3	− 1 to 14	7.1	1–13	7.3	1–12
Total learn	52.6	4–80	56.0	13–80	58.8	28–79	59.3	24–79	61.2	20–78	61.3	29–76
Del. recall*	11.6	0–16	12.2	0–16	13.1	0–16	13.6	3–16	14.1	4–16	14.1	5–16

Age and interval are given in years. Interval is interval since 1st visit. Hippocampus volume (Hip. vol.) denotes number of voxels (mm^y^) in the hippocampal segmentation bilaterally. Learn. Trial 1 = Learning trial 1; number of words recalled at first trial. Learn. slope = learning slope across five trials (trial 5 minus trial 1). Total learn. = total learning, the sum of words recalled across five consecutive trials. Del. Recall = delayed recall approximately 20 min after the short delay recall test that follows the 5 learning trials (delayed recall was missing for n = 7 at Timepoint (Tp) 1, n = 1 at Tp2, n = 2 at Tp3, and n = 1 at Tp5).

**Figure 1:**
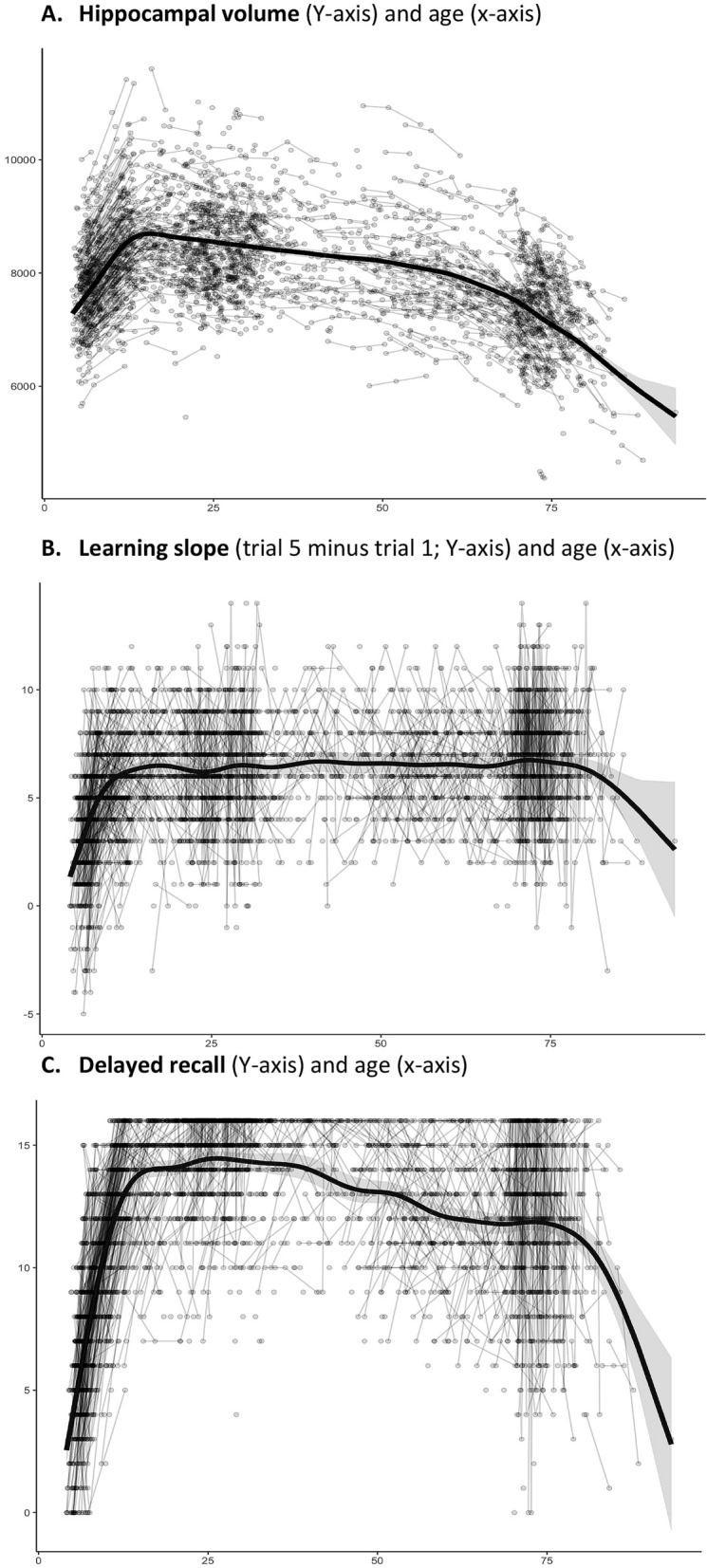
*Lifespan sample* changes in hippocampus, learning rates and memory. (**A**) Hippocampus volume (across hemispheres, shown in mm^3^ on the Y-axis) and change in relation to age (in years, x, axis) plotted with individual trajectories overlaid. (**B**) Learning slope (trial 5 minus trial 1 on the Y-axis) and change in relation to age (in years, x, axis) plotted with individual trajectories overlaid. (**C**) Delayed free recall of the 16 word list approximately 20 min after initial administration (Y-axis) and change in relation to age (in years, x, axis) plotted with individual trajectories overlaid. Shaded areas represent 95% confidence intervals.

In *the lifespan sample*, hippocampal volume, as shown in Fig. 1A, increased sharply in early development, reaching relative stability with a slight apparent decline shortly after the teenage years, until a sharper decline beginning around the age of 60 years, in line with previous research^19,20,50,51^. Learning slopes, as shown in Fig. 1B, increased sharply in early development, reached a plateau in teenage years, and then seemed to remain comparatively stable, with a slight apparent decline in the 70 s. Correlation analysis at time point 1, showed that learning slope was positively related to age in the *lifespan sample* (r = 0.24, p < 0.0001, df = 1823, CI 0.19, 0.28). Since we know that learning increases in development, remains relatively stable in adulthood, but shows sharper decrease in older adulthood^30,52,53^, we ran the same analysis in subsamples defined by age accordingly. These showed that the positive relationship was found only in development (age < 18 years; r = 0.51, df = 647, P < 0.0001, CI 0.45, 0.56), not in adulthood (age 18.00–69 years, r = 0.03, p = 0.0928, df = 1004, CI − 0.03, 0.09), and a negative relationship was found in the oldest participants (age ≥ 70 years, r = -0.27, p = 0.0004, df = 168, CI − 0.40, − 0.12). Delayed recall, as shown in Fig. 1C, also showed sharp developmental increase, with relatively greater stability, yet some continued improvement into the 20 s and 30 s, then slight decline, followed by an apparently sharper decline from the 70 s onwards.

Analysis with General Additive Mixed Models (GAMM) where delayed verbal recall was predicted from verbal learning slope, with first trial learning and total learning across five trials as covariates along with verbal learning test version, age as a smooth function, sex and subject timepoint, showed a significant positive effect of learning slope (t = 9.964, p < 0.0001, 3472 observations). The estimated effect of one unit (words) increase in learning slope on delayed recall was 0.248 increase in units (words) on delayed recall (CI 0.199, 0.297). Likewise, GAMM analysis where hippocampal volumes were predicted from verbal learning slope, with first trial learning and total learning across five trials as covariates along with verbal learning test version, age as a smooth function, sex, subject timepoint, intracranial volume and scanner as covariates, showed a significant positive effect of learning slope (t = 3.115, p = 0.0019, 3483 observations). The estimated effect of one unit increase in learning slope was 8.6 mm^3^ increase in hippocampal volume (CI 3.2, 14.1). For plots of these effects, see Supplementary Material (Supplementary Fig. 1A,B).

To further test whether including learning slope in these models provided additional explanatory power over and beyond models only including first trial learning and total learning, we compared models using the Akaike Information Criterion (AIC). These model comparisons showed that both in predicting delayed recall and hippocampal volume, including learning slope improved prediction in the *lifespan sample* (see Supplementary Material S1, Model Comparisons, p.2–4).

Individual differences in other abilities, including working memory, have been shown to affect verbal learning performance^54^. To illuminate whether effects of learning slope were specific, a measure of working memory which was administered in a comparable way across ages in the *lifespan sample*, digit span backwards, was added as an additional covariate in the above models. Results showed that also working memory was a significant predictor of delayed verbal recall (t = 3.866, p = 0.0001), but verbal learning slope remained a significant predictor when covarying also for working memory (t = 9.229, p < 0.0001, 3163 observations), confirming that the effect of learning slope on delayed recall could not be ascribed to more fundamental individual differences in working memory. The estimated effect of one unit (words) increase in learning slope on delayed recall was then 0.243 increase in units (words) on delayed recall (CI 0.191, 0.294). Working memory was not a significant predictor of hippocampal volume (t = 0.762, p = 0.4463), and when covarying for working memory, learning slope was still significantly related to hippocampal volume (t = 3.380, p = 0.0007, 3170 observations). The estimated effect of one unit increase in learning slope was 10.3 mm^3^ increase in hippocampal volume (CI 4.3, 16.3). To further investigate neuroanatomical specificity of effects, this analysis was next repeated now predicting putamen volume instead of hippocampal volume. Putamen was chosen because it is a subcortical structure of comparable size to the hippocampus, which is implicated in among other functions working memory^55^. Neither working memory (t = 0.159, p = 0.8733), nor learning slope (t = 0.224, p = 0.8230) were significant predictors of putamen volume. The estimated effect of one unit increase in learning slope was 1 mm^3^ increase in putamen volume (CI − 7.7, 9.6).

Since some floor and ceiling effects were unavoidable with a verbal learning test applied across an age range of 90 years in the *lifespan sample*, analyses were repeated excluding observations with a perfect recall score across all 5 trials, as well a first learning trial or delayed recall score of 0. Learning slope remained a positive predictor of both delayed verbal recall and hippocampal volume. Likewise, to further investigate robustness of results, analyses were repeated additionally excluding those enrolled in memory training intervention. Analyses restricted to adults were also performed. Learning slope remained a significant (p < 0.05) predictor of both delayed verbal recall and hippocampal volume in all these conditions (for details, see Supplementary Material S1, Sensitivity analyses in the *lifespan sample*, p.4–5).

Arguably, representing learning slope via a single difference score between trial 1 and trial 5, may not equally well capture the learning process across different stages of the lifespan. As seen from the descriptive figures and correlation analyses in the present *lifespan sample*, learning slope correlated positively with age in development only, while a negative correlation was observed in aging. Differences in learning curves of children and adults have been reported previously, using more complex models with other data^37^. To provide series of different slope estimates for different subsets of the lifespan is beyond the scope of this paper. However, we also described the learning slopes/curves with principal components in the *lifespan sample* (see Supplementary Material S1, Describing the learning slopes using principal components, p. 5–8). Four principal components are sufficient to completely describe learning across the five trials, and our results showed that using the first two of these components as replacements for the difference score in the GAMMs gave meaningful interpretations. Both for prediction of hippocampal volume and for prediction of 30-min free recall, the first principal component was of highest importance, and further analyses revealed that this first component very closely resembles that difference score between the last and the first timepoint. Hence, this alternative method of modeling^56^ the learning curves supports our interpretation of the main results.

While learning slope explains unique variance in hippocampal volume in a mixed model, this does not necessarily imply that change in learning slopes longitudinally is coupled with hippocampal change. To assess the change-change correlation in the *lifespan sample*, we excluded timepoints with less than 6 months between them and excluded participants with a single timepoint, and then computed two GAMMs independently, having learning slope and hippocampal volume as dependent variables, respectively, as smooth functions of age. Sex was included as an additional covariate for both models, while scanner and ICV were included as covariates in the model for hippocampal volumes. Both GAMMs had random slopes for age, showing for each person whether he or she tended to change more positively or more negatively than average, given his/her age. Correlated change in learning slope and hippocampal volume was computed as the Pearson correlation between each person's random slopes in the two models. The Pearson correlation for hippocampal change and change in learning was 0.01 (CI − 0.06, 0.08; t = 0.3260, df = 683, p = 0.7445). The correlation was additionally computed within development (< 18 years of age), adulthood (18–69 years of age) and older adulthood (age ≥ 70 years). In no case was the change-change correlation significant, neither in development (r = 0.04, CI − 0.071, 0.146), nor adulthood (r = − 0.09, CI − 0.197, 0.018) or older adulthood (r = 0.28, CI − 0.07, 0.59), although we note that the correlation appeared somewhat stronger in older adulthood.

### Analyses in the training sample: within-session learning slope is positively related to benefit from memory training across 10 weeks

**Figure 2:**
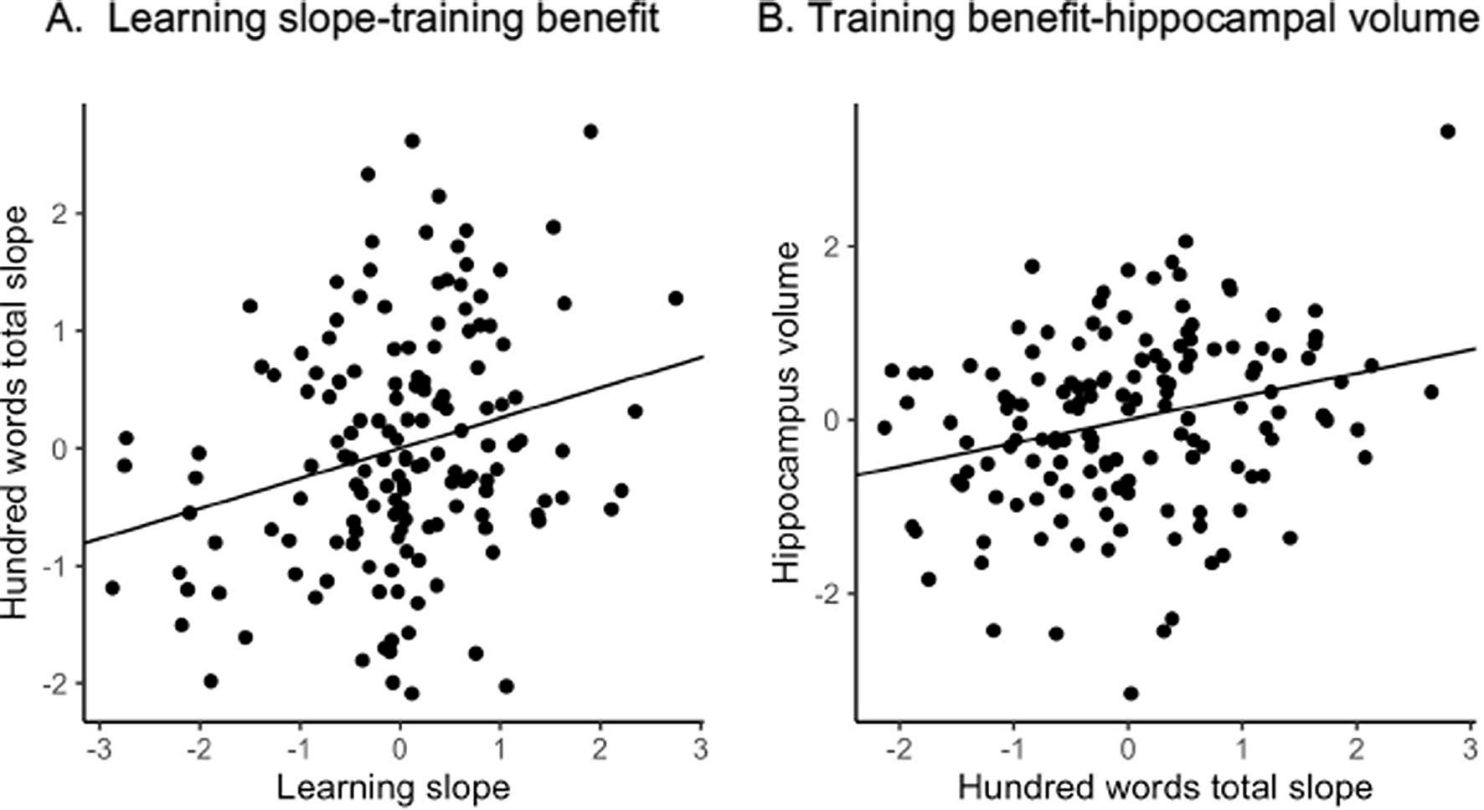
*Training sample* relationships of benefit from ten-week memory training. (**A**) Memory training benefit standardized scores (y-axis, 100 word test performance post- minus pre ten weeks of memory training) and within-session learning slope standardized scores (x-axis), residual values after partialling out offset scores and interval between first verbal learning test and 100 words-pre-training test, and (**B**) Hippocampus volume standardized values (across hemispheres, y-axis), and memory training benefit standardized scores (x-axis, 100 word test performance post- minus pre ten weeks of memory training), residual values after partialling out age, sex, 100-words baseline score, the interval between scan and 100 words-pre-training test, and intracranial volume.

A subsample (n = 155, Neurocognitive Plasticity (NCP) study, see “Methods”) of adults in the lifespan sample underwent strategic episodic memory training after one or more MRI scan and cognitive testing. This is here termed the *training sample*. To avoid ceiling effects, benefit from verbal memory training was measured by a 100-word recall test at baseline and after a period of 10 weeks of training. We partialled out offset scores (verbal learning test first trial score and 100-word recall test pre-training), interval between first verbal learning test and 100-word-pre-training test, sex, and age (see ““Memory tests”, and Supplementary Material S1, Information on the Memory training program (NCP) for further information). In the *training sample*, single session learning slope at baseline pre-training correlated positively with memory training benefit across 10 weeks of training (r = 0.25, p = 0.0020), showing that within-session learning slope related positively to long-term cognitive plasticity in terms of strategic memory training benefit. The relationship between within-session learning slope and benefit from ten-weeks training, regardless of age, sex and memory score intercepts, is shown in Fig. 2. Benefit from memory training also correlated positively with hippocampal volume at baseline within this sample (n = 153), when partialling out age, sex, 100-word baseline score, the interval between scan and 100-word-pre-training test, and intracranial volume (ICV) (r = 0.26, p = 0.0015). This correlation in the *training sample* demonstrates the positive relationship between hippocampus and learning capacity also across weeks.

### Analyses in the twin sample: genetic influences on learning slope

**Figure 3:**
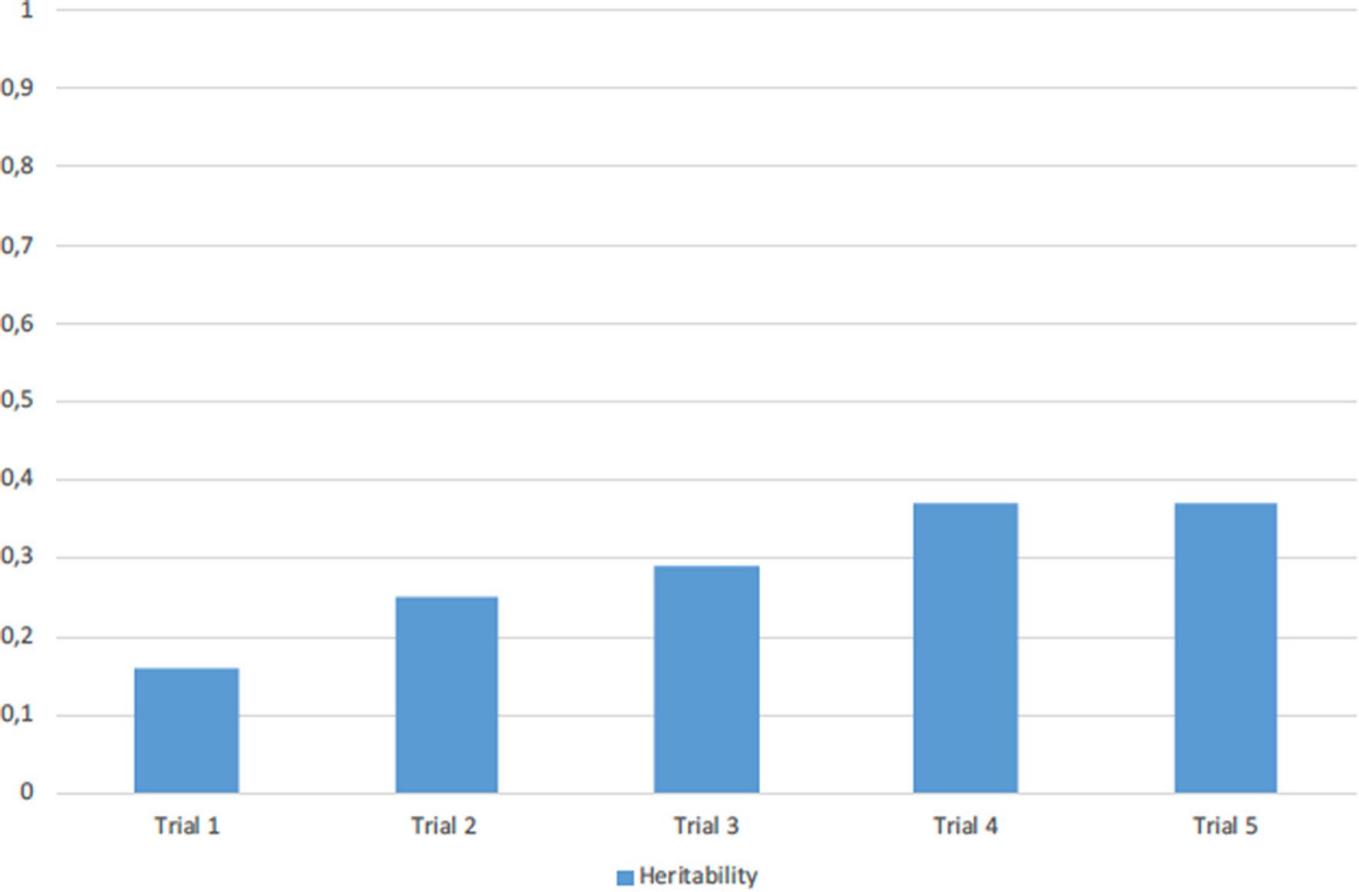
*Twin sample* heritability of each of the California Verbal Learning Test trials. Bars show the relative contribution of genetic influences across the individual learning trials, going from a heritability of 0.16 (95% CI 0.03–0.31) at trial 1 to a heritability of 0.37 (95% CI 0.19–0.46) at trial 5.

The heritability of learning slope was investigated in The Vietnam Era Twin Study of Aging (VETSA) (n = 1240 male twins aged 51–60 years), here called the *twin sample*. Across the individual learning trials the relative contribution of genetic influences gradually increased, going from a heritability of 0.16 (95% CI 0.03–0.31) at trial 1 to a heritability of 0.37 (95% CI 0.19–0.46) at trial 5 (see Fig. 3). Supplementary Fig. 2 shows the phenotypic variance and the relative contributions of genetic and environmental variance across the learning trials. Notably, as seen here, phenotypic variance also increased across trials in the twin sample. Although the heritability estimates for trial 1 and trial 5 differed substantially, they were not significantly different from one another based on the 95% confidence intervals. In a genetically informed linear growth curve model, learning slope showed significant heritability of 0.44 (95% CI 0.15–0.71). While the relative contribution of genetic influences was roughly equivalent to the heritability of the intercept (i.e. first trial) factor (0.47, 95% CI 0.24–0.61), the absolute genetic variances for intercept and slope differed markedly: 1.20 for the intercept and 0.05 for slope. This discrepancy was consistent with the observed phenotypic variances of the two factors (2.55 for intercept and 0.12 for slope). The genetic correlation between intercept and slope, indicating the degree of genetic overlap between the two, was 1.0, indicating that the genetic factors underlying learning slope are identical to those that influence performance at any individual learning trial. This should be interpreted in light of the phenotypic correlation between intercept and slope, which was significant, but relatively moderate (r = − 0.28, p < 0.001, for CVLT trial 1 and learning slope).

## Discussion

We found that within-session verbal learning slope, i.e. ability to benefit from practice across repeated episodic memory trials, uniquely explained variance in both delayed recall and hippocampal volume across the lifespan. The variance explained by verbal learning slope was beyond what was explained by performance after a single learning trial, the aggregate performance across trials, estimated working memory capacity, age, and sex. This finding highlights short-term learning benefit per se as metric of high interest. We further showed that this short-term learning benefit, within a session, related positively to benefit from memory training across weeks, demonstrating a broader relevance. Finally, we demonstrated significant heritability of individual differences in learning slope, and while genetic influences on slope appeared to be the same as those on intercept, performance after multiple learning trials tended to a greater extent to reflect differences in genetic variance.

Given the unique value shown of the slope of learning in healthy persons of different ages, it is unfortunate that research has chiefly focused on single measures of recall^28^. The present findings indicate that practice may promote prediction, in that the learning slope measure explained unique variance in delayed recall beyond that explained by single trial learning or aggregate performance across trials. In a recent cross-sectional study of older adults, it was found that the interaction of hippocampal volume and limbic white matter diffusivity characteristics, but not hippocampal volume alone, predicted verbal learning rates^39^. In that older population, greater hippocampal volume was only associated with better learning rate in older adults with higher fractional anisotropy in limbic regions^39^, and not in those with presumably lower white matter integrity. We cannot perform the same analyses with the current cognitively healthy lifespan sample which is based on macrostructural scans, and what white matter anisotropy signifies also likely differs across stages of the lifespan. However, the present findings extend previous observations in a cognitively healthy lifespan population, with unique relations of learning slope to delayed recall and hippocampal volume.

Moreover, the establishment of a relationship between short-term learning capacity and benefit from long-term cognitive training may serve as an indication that within-session measures of plasticity can be utilized as a marker of long-term potential for modifying performance. As long-term cognitive training studies are costly and time-consuming, within-session measures of learning may then be a marker of substantial interest in selection of individuals for cognitive training interventions and trials.

The significant heritability of learning slope in the current twin sample, support the notion that also higher order short-term cognitive plasticity is heritable, as previously indicated for basic motor learning or conditioning in twin studies of practice effects. As reported for acquisition of skill for rotary pursuit^34^, heritability appeared to increase with number of trials for verbal learning. A previous twin study of motor evoked potential implicated that genetic factors may contribute significantly to inter-individual variability in plasticity paradigms^36^, with a derived heritability estimate of 0.68. The present study shows that such genetic influences can be found also on training of a higher-order cognitive function, episodic memory, even if the heritability estimate of higher order learning found here may be slightly lower (0.44 for learning slope). While the present results indicate that the genetic influences on variance in slope are similar as to those for offset (first trial score), they also underscore that the relative proportion of variance in episodic memory being grounded in genetic variance may be heightened with practice. This finding should be of interest to both the basic science field, intervention and education.

## Limitations and future directions

There are limitations to the present study that should be addressed by future research. First, the application of a similar list learning test across the lifespan yielded some floor and ceiling effects. While results remained unchanged when excluding performers with bottom and top scores in the lifespan sample, development and validation of new tests with adaptive difficulty level may yield improved measures of learning slope across the lifespan. It should also be noted that the verbal learning tests used here were designed with four semantic categories, and results may not be directly transferable to other popular verbal learning tests such as the Rey Auditory Verbal Learning Test^57^ or the Hopkins Verbal Learning test—Revised^58^ used in other studies. It is also evident that since the learning material used is verbal, effects may be limited to verbal learning, and cannot necessarily be generalized to other modalities. Also, we used a readily available and simple measure of short term learning slope, and more complex and detailed measures, such as items touched and turnover may reveal differences across the lifespan^59^. Since education was not collected in a comparable manner across all participants, the relations to education could not be investigated. As cognitive training samples, including the present, typically are not very large, we did not have the possibility to replicate findings in separate subsamples. Future studies are needed to investigate further whether learning slope may be useful e.g. in the selection of participants for training studies. Furthermore, the participants in all studies were cognitively healthy. Differences in learning rate have been reported in clinical groups^60^, and future studies should investigate to what extent short-term learning slope posit the same relations to hippocampal characteristics, long-term learning benefit and genetic influences in clinical groups. It should also be noted that heritability of learning slope was only evaluated in middle aged men, and this needs to be studied also across sex and ages.

## Conclusions

These results indicate that extent of plasticity of memory, i.e. short-term rate of verbal learning, has unique predictive value for long-term hippocampal characteristics and training benefits across the human lifespan, and is heritable. This calls for memory plasticity, beyond performance per se, as an important biomarker through the lifespan.

## Methods

All methods were carried out in accordance with relevant guidelines and regulations, including the Declaration of Helsinki.

### Lifespan and memory training samples

For these samples, the studies were approved by the Regional Ethical Committee of South East Norway https://helseforskning.etikkom.no/komiteerogmoter/sorost/sekretariat?region=10795&p_dim=34981&_ikbLanguageCode=us . Informed consent was obtained for all participants; in writing from those 16 years and older, and from parents of participants below 16 years of age. (In Norway, where the data from the young participants was collected, the age of majority for health-related questions is 16 years of age.) Participants 12 years and older also gave oral consent.

### Lifespan sample: LCBC

Participants in the lifespan sample (1) were cognitively healthy community-dwelling volunteers. A total of 3483 valid scans and combined test session data from 1825 cognitively healthy participants, 4.1–93.4 years of age (mean visit age = 36.5 years, SD = 25.1 years), were drawn from four Norwegian sub-studies coordinated by the Center for Lifespan Changes in Brain and Cognition (LCBC); The Norwegian Mother and Child Cohort Neurocognitive Study (with partcipants recruited from the Norwegian Mother, Father and Child Cohort study, MoBa, at the Norwegian Institute of Public Health, see)^61^, Neurocognitive Development (ND)^62^, Cognition and Plasticity Through the Lifespan (CPLS)^63^, Neurocognitive Plasticity (NCP)^64^. MoBa, ND, and CPLS were observational studies. However, within the CPLS sample, 22 persons, with a total of 41 observations, were offered 8 weeks of memory training memory as a precursor study to the NCP study, but without the same memory training benefit measure (100 words test, see below). Separate analyses were thus conducted with and without these observations. In the NCP study, all were offered one or two 10-week periods of memory training or rest, with scans in between these. All persons in the NCP project were offered memory training at some time point, and separate analyses were thus conducted without these, as well as with these only (see below for separate description of NCP procedures). The majority of participants in the lifespan sample were followed longitudinally, with intervals ranging 0.2–11.0 years (mean = 3.1 years, SD = 2.7 years). The sample is partly overlapping with^65,66^. Education was initially recorded somewhat differently across sub-projects, but for most, education was recorded as number of years of education to the highest attained degree for adults (age ≥ 18 years), and for participants below 18 years of age, the average of paternal and maternal years of education to the highest attained degree was entered, or if unavailable, for one parent (either available). By this measure, education was obtained in a comparable manner for most participants (n = 1253, mean = 16.1 years, SD = 2.7 years, range 8–23 years at first timepoint; this education was collected for additional participants later on, in total n = 1376, mean = 16.3, SD = 2.7, range 8–23 years). Dementia, previous stroke with sequela, Parkinson’s disease, and other neurodegenerative diseases likely to affect cognition were exclusion criteria across all projects, with additional inclusion and exclusion criteria being applied per study.

Participants above 60 years of age were required to have a Mini mental Status Examination^67^ score ≥ 26 to be included in the present analyses. Complete absence of health problems was not required for inclusion. Participants with common health conditions, such as moderately elevated blood pressure and being on antihypertensive treatment, were not excluded. They were recruited in part by newspaper and online adverts, and in part through the population registry cohort study MoBa^61^. Additional criteria for being included in the present analyses were (1) having data recorded for all five learning trials for CVLT, and (2) having a valid anatomical MRI scan with successful automatic hippocampal segmentation (see below). Sample descriptions for the total sample binned by timepoints are given in Table 1. Additional descriptions including distribution of sub-study samples per timepoint are given in Supplementary Table 1. All participants were compensated a modest sum for their participation, depending on amount of examinations (for the structural scan session around NOK 500, or USD $60).

### Training sample: neurocognitive plasticity (NCP)

The sample was a subsample of the lifespan sample described above, who took part in memory training in the project Neurocognitive Plasticity at the Center for Lifespan Changes in Brain and Cognition (LCBC), Department of Psychology, University of Oslo. The final sample for this analysis after application of exclusion criteria (see below) consisted of a group of younger (n = 56, of whom 30 females, mean age = 26.4 years, SD = 3.1 years, range = 20.5–31.1 years), and older adults (n = 99, of whom 63 females, mean age = 73.3 years, SD = 3.0 years, range = 69.0–81.9 years). Mean years of education for the sample was 15.4 years (SD = 2.7 years, range 7.5–21 years). Participants were recruited through newspaper and web page adverts and were screened with a health interview. Participants were required to be either young or older (in or around their 20 s or 70 s, respectively) healthy adults, right-handed, fluent Norwegian speakers, and have normal or corrected to normal vision and hearing. Exclusion criteria were history of injury or disease known to affect central nervous system function, including neurological or psychiatric illness or serious head trauma, being under psychiatric treatment, use of psychoactive drugs known to affect central nervous system functioning, and MRI contraindications. Moreover, for inclusion in the present study, participants were required to score ≥ 26 on the Mini-Mental State Examination (MMSE)^68^ and have scores within normal range (≥ 2 standard deviations below mean) for age and sex on the 5-min delayed recall subtest of the California Verbal Learning Test II^30^. All participants further had to achieve an IQ above 85 on the Wechsler Abbreviated Scale of Intelligence^69^. Three participants in the older group were excluded based on these criteria. Participant scans were evaluated by a neuroradiologist and deemed free of significant injuries or conditions. The images were further manually quality checked for artifacts affecting segmentation, and one older participant was excluded from the MRI analysis due to scan artifacts, while another had missing MRI data, reducing the sample for hippocampal volume relations to n = 153. Only participants who underwent follow-up assessment after memory training and neuropsychological tests at baseline were included in the current analyses. A total of 25 participants (11 young, 14 older) dropped out before the follow-up session, and were thus excluded from the longitudinal plasticity analyses. The participants who dropped out reported that the participation was too time consuming or that the particular time frame for assessment was inconvenient. Additionally, memory test data for time point 1 was lacking for one participant, who was hence excluded from the present analyses. For details of the memory training program, see Supplementary material S1 and^70^.

### MRI data acquisition

Participants were scanned at a total of 4 S scanners at 2 sites (1: Oslo University Hospital, Oslo, 2: St.Olav´s Hospital, Trondheim): A 1.5 T Avanto equipped with a 12 channel head coil (Site 1 and 2), a 3 T Skyra equipped with a 24-channel Siemens head coil (Site 1) or a 3 T Prisma equipped with a 32 channel head coil (Site 1) (all Siemens Medical Systems, Erlangen, Germany). Our MRI data acquisition methods are also described elsewhere, see^19,65,66^. The pulse sequence used for morphometric analyses were one to two 3D sagittal T1-weighted MPRAGE sequences. Avanto site 1 and 2: 160 slices, repetition time (TR), 2400 ms; echo time (TE), 3.61 ms/3.79 ms (Site 1/2); time to inversion, 1000 ms; flip angle, 8**°**; matrix, 192 × 192; field of view, 240; voxel size, 1.25 × 1.25 × 1.20 mm per participant per visit. Scanning time for each MPRAGE sequence was 7 min 42 s. For the children recruited for the MoBa study we used a parallel imaging technique (iPAT), using the same scan parameters, acquiring multiple T1-scans within a short scan time (acquisition duration of 4 min 18 s.), enabling us to discard scans with residual movement.

Skyra: 176 slices, TR = 2300 ms, TE = 2.98 ms, flip angle = 8°, voxel size = 1 × 1 × 1 mm, FOV = 256 × 256 mm. Prisma: 208 slices, TR = 2400 ms, TE = 2.22 ms, TI = 1000 ms, flip angle = 8°, voxel size = 0.8 × 0.8 × 0.8 mm^3^, FOV = 240 × 256 mm^2^. Other MRI volumes were recorded including sequences intended for and examined by a radiologist, to rule out and medically follow up incidental neuroradiological findings. Distribution of scans from the different scanners per timepoint is given in Supplementary Material (please see Supplementary Table 2).

### Image analysis

All scans were reviewed for quality and automatically corrected for spatial distortion^71^. Images were first automatically processed cross-sectionally for each time point with the FreeSurfer software package (version 6.0). This processing includes motion correction, removal of non-brain tissue, automated Talairach transformation, intensity correction and automatic volumetric segmentation, including hippocampal volumetric segmentation^72,73^. In older subjects, FreeSurfer is shown to calculate consistent hippocampal volumes with reproducibility errors of 3.4–3.6%^74^. While FreeSurfer has previously been shown to yield higher volume estimates than manual segmentation, particularly in younger than older adults^75^, these biases have been shown to be weaker in version 6.0 than in previous versions^76^.To extract reliable longitudinal subcortical volume estimates, the images were run through the longitudinal stream in FreeSurfer^77,78^. Specifically, an unbiased within-subject template volume based on the cross-sectional images was created for each participant, and processing of all time points was then initialized using common information from this template. This increased sensitivity and robustness of the longitudinal analysis and ensured inverse consistency^77^. In addition, new probabilistic methods (temporal fusion) were applied to further reduce the variability across time points. Participants followed-up on different MRI scanners were independently processed for each scanner. To allow assessment of differences between scanners, 24 participants were scanned on all three scanners from Oslo University Hospital on the same day. Linear regression analyses were run testing the concordance between hippocampal volumes between scanners, yielding excellent agreement (Avanto vs Prisma R^2^ = 0.93; Prisma vs Skyra R^2^ = 0.94; Prisma vs. Avanto R^2^ = 0.90). Thus, including scanner as covariate in the analyses would almost perfectly account for any possible scanner bias.

### Twin sample: the Vietnam era twin study of aging (VETSA)

The VETSA is a longitudinal study of cognitive and brain aging, with baseline in midlife (mean age = 55.9, SD = 2.4, range 51.1–60.7). Memory data was initially available from 1291 participants. Of these, fifty-one cases were excluded due to errors in test administration, or because participant reported a history of stroke or other brain disease (e.g., brain cancer). All participants gave written informed consent to be in the study. The study protocol was approved by the Institutional review boards of the participating institutions, the Human Research Protections Program of the University of California, San Diego https://irb.ucsd.edu/Home.FWx , and the Boston Medical Center and Boston University Medical Campus Institutional Review Board http://www.bumc.bu.edu/irb/. Data for this study were collected at 2 sites: University of California, San Diego, and Boston University. For the 1240 men included in the present analysis, mean level of education was 13.8 years (SD = 2.1; range 8–20). Memory analyses included 338 monozygotic (MZ) and 254 dizygotic (DZ) twin pairs and an additional 56 unpaired twins from two sites (see Supplementary Material S1).

### Memory tests

Different versions of the California Verbal Learning Test (CVLT)^30,52,79^ were used across subprojects of the Lifespan sample and the VETSA sample. The standard CVLT administration procedure, with a list of 16 words read to the participant over five trials was followed, with the exception of children below 6.5 years, where the list was reduced to 12 words. After each trial the participant was asked to repeat all of the words she/he could remember. Following these initial learning trials an interference list was read, and the participant was then asked to recall all of the words from the new list as possible. The interference list was followed by a short delay free recall of the first list (“5 min recall”). Approximately 20 min later there was a long delay free recall of the first list (delayed recall). Directly following the short and long delay free recall conditions, a cued recall condition was administered in which the participant was prompted with the four semantic categories of the words on the first list (in the reduced 12 word version for children, one word was subtracted from each category, so categories were retained). In the VETSA sample, the CVLT II original version was used. Different Norwegian versions of the CVLT, including the CVLT I, CVLT II original and alternate version^30,52,79^, were used in different follow-up waves of the lifespan sample, to reduce direct recall effects across testing occasions. In addition, two additional word list tests were created, mimicking the exact structure of the CVLT, with 16 words across 4 different semantic categories, were created and employed, given the multitude of follow-ups. This yielded 5 different word list tests, all used in NCP (for participants completing all 6 follow-ups here, the original CVLT II version was used at TP2 and Tp6). On the majority of testing occasions, CVLT II was used, in either the original (1311 occasions) or alternate version (1185 occasions), of which a small proportion involved testing children below 6.5 years, and hence administration of the simplified version of these (249 occasions in total). CVLT 1 was administered on 559 occasions, and a created version on a total of 427 occasions. Test version was controlled for in analyses (see “Statistical analyses”).

Learning slope, calculated as trial 5 minus trial 1 score, was used as measure of learning slope. The first trial learning score (the “offset”) as well as the total of trials 1 through 5, i.e. total learning, were used as covariates in analyses. The long delay free recall condition was utilized as indicator of delayed verbal recall.

In the memory training sample, NCP, benefit from memory training was measured by change in correct written recall of a word list consisting of 100 nouns administrated in the laboratory on the neuropsychological test sessions on baseline and after the intervention, on the follow-up test. The task measured change in correct written recall of a word list consisting of 100 nouns, in terms of number of nouns remembered (regardless of serial position), that is the score post-intervention minus baseline score. The participants were given five minutes to memorize the word list, followed by ten minutes to recall as many words as possible. The words in the lists differed between the two time points. The extensive length of the word lists was chosen to avoid ceiling effects. For more details regarding memory training program and the individual adjustments, see Supplementary Material S1^70^.

### Statistical analyses

Analyses were run in R^80^ version 3.6.1*.* General Additive Mixed Models (GAMM) using the package “mgcv”^81^ version 1.8–28 were used to derive age-functions with a random intercept term per participant. Delayed verbal recall was predicted from a linear function of verbal learning slope, with a smooth function of age, and linear functions of sex and first trial learning and total learning across five trials as covariates, along with verbal learning test version and subject timepoint (to account for effects of repeat test administrations throughout the follow-up period). Hippocampal volumes were predicted from a linear function of verbal learning slope, with a smooth function of age, and linear functions of verbal learning test version, sex, scanner, intracranial volume, first trial learning and total learning across five trials as covariates, along with subject timepoint. Marginal maximum likelihood was used for smoothness selection.

To test the hypothesis that rate of learning as assessed within a single session is positively related to plasticity as assessed in long-term memory training, we correlated within-session learning slope (first administration of CVLT) and plastic changes across 10 weeks in the experimental training sample (sample 1) undergoing extended memory training. Age, sex and baseline scores (CVLT trial 1, 100 words test score pre-training) were partialled out, along with interval between first administration of CVLT and the pre-training 100 words test score. To check whether plasticity as assessed in long-term memory training was also related to hippocampal volume, we correlated change in 100 words recall after 10 weeks memory training (100 words post-training test–100 words pre-training test) with hippocampal volume at first MRI. Age, sex and 100 words test score pre-training were partialled out, along with interval between first MRI and the pre-training 100 words test score. Procedures for additional follow-up analyses are described in the Results section and in Supplementary Material S1.

To test the hypothesis that learning slope was heritable and that heritability increased with learning, multivariate biometric analyses were performed using the structural equation modelling software OpenMX^82^. A multivariate Cholesky decomposition model was first fit to the data in order to estimate the genetic and environmental variances and covariances among the learning trials. The Cholesky also allowed us to estimate the heritability (i.e., the standardized genetic variance) at each trial. The genetically informative latent growth curve model was fit using a variant of the widely used common pathway or psychometric factors model^83^. In this model, two latent factors were fit to the data, representing intercept and linear slope. Loadings for the intercept factor were fixed at 1.0, while loadings of the slope factor were fixed at − 2, − 1, 0, 1, and 2 (centering the data at trial three). Residual variance of each observed variable was constrained to be equal, and the model was specified so that only unique environmental factors could contribute to the residual variance. The variances of the intercept and slope factors were decomposed into additive genetic (A), common environmental (C), and unique environmental (E) variance components, and the latent factors were allowed to correlate at genetic and environmental levels.

## Supplementary information

Supplementary Information

## Data Availability

Data not published are subject to restricted access due to ethical and data protection legislation, but will be made available pending reasonable request, appropriate institutional data protection security measures, and pending ethical approval.
