# Supplementary material for: Within-session verbal learning slope is predictive of lifespan delayed recall, hippocampal volume, and memory training benefit, and is heritable
Source: Sci Rep. 2020 Dec 3;10:21158. doi: 10.1038/s41598-020-78225-1 (PMC7713377; doi:10.1038/s41598-020-78225-1)
Supplement: Supplementary file 1 — Supplementary Information [file 41598_2020_78225_MOESM1_ESM.docx]

**Supplementary Material**

For

**Within-session verbal learning slope is predictive of lifespan delayed recall, hippocampal volume, and memory training benefit, and is heritable**

Kristine B Walhovd, Anne Cecilie Sjøli Bråthen, Matthew S. Panizzon, Athanasia M. Mowinckel, Øystein Sørensen, Ann-Marie de Lange, Stine Kleppe Krogsrud, Asta Håberg, Carol E. Franz, William S. Kremen, Anders M. Fjell

**Supplementary Results**

**Supplementary Table 1 Relationships between variables of interest.**

Pearson correlations at time point 1 are shown. All correlations are significant at p <. 05. This Table is shown for descriptive purposes only.

|  | **Learning trial 1** | **Total learning** | **Learning slope** | **Delayed recall** | **Hippo. volume** | **Age** |
| --- | --- | --- | --- | --- | --- | --- |
| **Learning trial 1** | ___ | .80 | -.19 | .64 | .20 | .05 |
| **Total learning** | ___ | ___ | .38 | .88 | .26 | .16 |
| **Learning slope** | ___ | ___ | ___ | .45 | .13 | .24 |
| **Delayed recall** | ___ | ___ | ___ | ___ | .26 | .20 |
| **Hippo. volume** | ___ | ___ | ___ | ___ | ___ | -.23 |


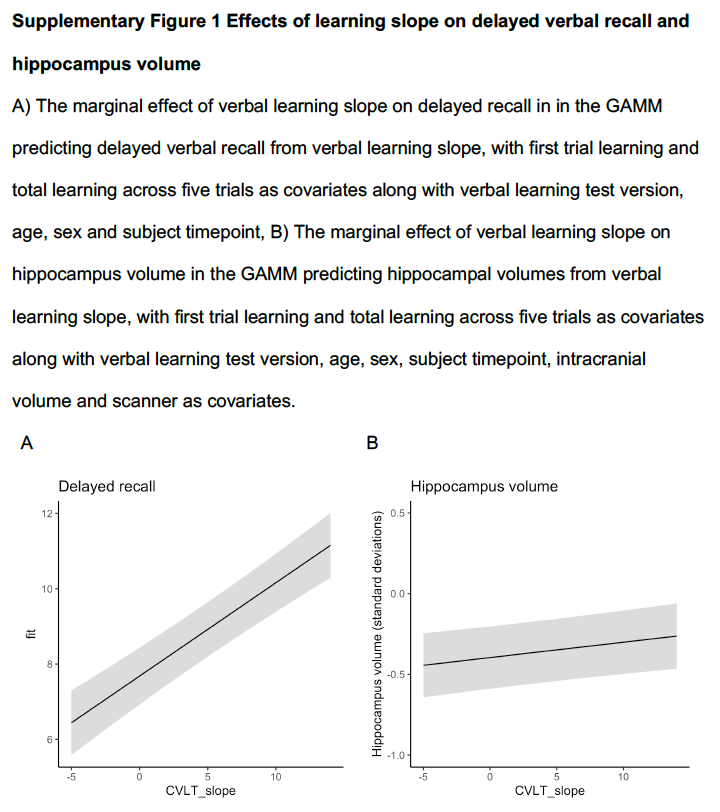


*Model comparisons*

Using the Akaike Information Criterion (AIC), we compared 4 models for predicting delayed recall:

1. Model without any memory score predictors, including

Age (smooth function) + Sex + Memory test version + Subject time point

1. Model with first trial learning and total learning, including

Age (smooth function) + Sex + Memory test version + Subject time point + First trial learning + Total Learning

1. Model with first trial learning, total learning, and learning slope, including

Age (smooth function) + Sex + Memory test version + Subject time point + First trial learning + Total Learning + Learning Slope

1. Model with learning slope as only memory score, including

Age (smooth function) + Sex + Memory test version + Subject time point + + Learning Slope

In terms of minimizing AIC, the model with first trial learning, total learning, and learning slope (model 3) is best:

Model 1, df = 13, AIC: 6940.880

Model 2, df = 15, AIC: 4546.984

Model 3, df = 16, AIC: 4444.715

Model 4, df = 14, AIC: 6808.132

Again, using AIC, we compared 4 models for predicting hippocampal volume:

1. Model without any memory score predictors, including

Age (smooth function) + Sex + ICV + scanner

1. Model with first trial learning and total learning, including

Age (smooth function) + Sex + ICV + scanner + First trial learning + Total Learning

1. Model with first trial learning, total learning, and learning slope, including

Age (smooth function) + Sex + ICV + scanner + First trial learning + Total Learning + Learning Slope

1. Model with learning slope as only memory score, including

Age (smooth function) + Sex + ICV + scanner + Learning Slope

In terms of minimizing AIC, the model with first trial learning, total learning, and learning slope (model 3) is best:

Model 1, df = 10, AIC: 2921.899

Model 2, df = 12, AIC: 2923.579

Model 3, df = 13, AIC: 2917.310

Model 4, df = 11, AIC: 2917.466

As seen from the AICs, in predicting both delayed recall and hippocampal volume, the models, including learning slope in addition to frist trial learning and total learning, performed the best.

*Sensitivity analyses in the lifespan sample*

Analyses were repeated excluding observations with a perfect recall score across all 5 trials (3 observations), as well a first learning trial or delayed recall score of 0 (31 observations in total, mostly very young children, median age = 5.5 years). Learning slope remained a positive predictor of both delayed verbal recall (t = 9.595, p <.0001, 3438 observations) and hippocampal volume (t = 2.896, p = .0038, 3438 observations). The estimated effect one unit (words) increase in learning slope on delayed recall was then 0.233 increase in units (words) on delayed recall (CI: .185, .281), while the estimated effect of one unit increase in learning slope was then 8.1 mm^3^ increase in hippocampal volume (CI: 2.6, 13.6). Analyses were repeated without the sample for the memory training project NCP, and those in CPLS who took part in memory training, and excluding observations with a perfect learning across all 5 trials, as well a first learning trial or delayed recall score of 0. Learning slope remained a positive predictor of both delayed verbal recall (t = 7.960, p <.0001, 2514 observations) and hippocampal volume (t = 1.970, p = .0490, 2514 observations). Analyses were next repeated restricting the sample to adults only (age >= 18.00 years). Learning slope remained a positive predictor of both delayed verbal recall (t = 10.962, p <.0001, 2423 observations) and hippocampal volume (t = 2.203, p = .0277, 2427 observations). The estimated effect one unit (words) increase in learning slope on delayed recall was then 0.304 increase in units (words) on delayed recall (CI: .250, .358), while the estimated effect of one unit increase in learning slope was then 6.8 mm^3^ increase in hippocampal volume (CI: 0.8, 12.9).

*Describing the learning slopes in the lifespan sample using principal components*

Since each assessment consists of five trials, the learning, interpreted as the change through the trials without consideration of the total score, is fully described by four principal components. As an alternative to the difference score between the last and the first trial, these principal components can be used as explanatory variables in a regression. For each participant-timepoint we subtracted the mean across the five trials from the raw scores, yielding a matrix of rank four. From this matrix, four principal components were computed, representing a transformation of the learning curves with no loss of information. This analysis amounts to representing each participant’s learning curve as a weighted sum of four “characteristic shapes”, represented by the four components. Using these as explanatory variables in a GAMM may hence help interpreting which characteristics of the learning curve explain variation in hippocampal volume and 30-minute free recall.

The proportion of variance explained by each principal component is summarized below.

|  | Comp. 1 | Comp. 2 | Comp. 3 | Comp. 4 |
| --- | --- | --- | --- | --- |
| Proportion of variance | 0.48 | 0.24 | 0.15 | 0.13 |
| Cumulative proportion | 0.48 | 0.72 | 0.87 | 1.00 |

Both for the GAMM predicting 30-minute free recall and for the GAMM predicting hippocampal volume, we replaced the slope variable with one or more principal components, while keeping the remaining model structure unchanged.

For prediction of hippocampal volume, use of the first two principal components was optimal in terms of minimizing AIC. Their regression coefficients are shown in the table below. The estimates are in units of mm^3 per CVLT score.

| Component | Estimate | 95 % CI | p-value | t-statistic |
| --- | --- | --- | --- | --- |
| 1 | -3.66 | -7.05; -0.26 | 0.031 | -2.16 |
| 2 | 4.13 | -0.37; 8.63 | 0.066 | 1.84 |

Hence, component 1 appears to have a negative effect on hippocampal volume and component 2 appears to have a positive effect. The figure is intended to help with interpretation of these effects. Note that the offset of the curves is arbitrary, since the total CVLT score is not included. The plot can be read as follows.

- Component 1: A subject whose value along the first principal component axis corresponds to the 25^th^ sample percentile is characterized by a steep increase across trials, compared to subjects with a higher value of this component, as can be seen be the flattening of the curves for subjects with higher values of this component. Hence, the negative regression coefficient for this component implies that a low value of the component, which is characterized by a steep learning effect, is associated with higher hippocampal volume. Finally, note that component 1 to a large extent describes the difference between the score in the last and the first trial.
- Component 2: A subject whose value along the second principal component axis corresponds to the 25^th^ sample percentile is characterized by a learning effect that levels off after the first three trials. In contrast, a higher value of this component is associated with continuing learning throughout the trials. Hence, the positive regression coefficient for this component implies that a learning effect that does not level off but instead increases throughout the trials, is associated with higher hippocampal volume.


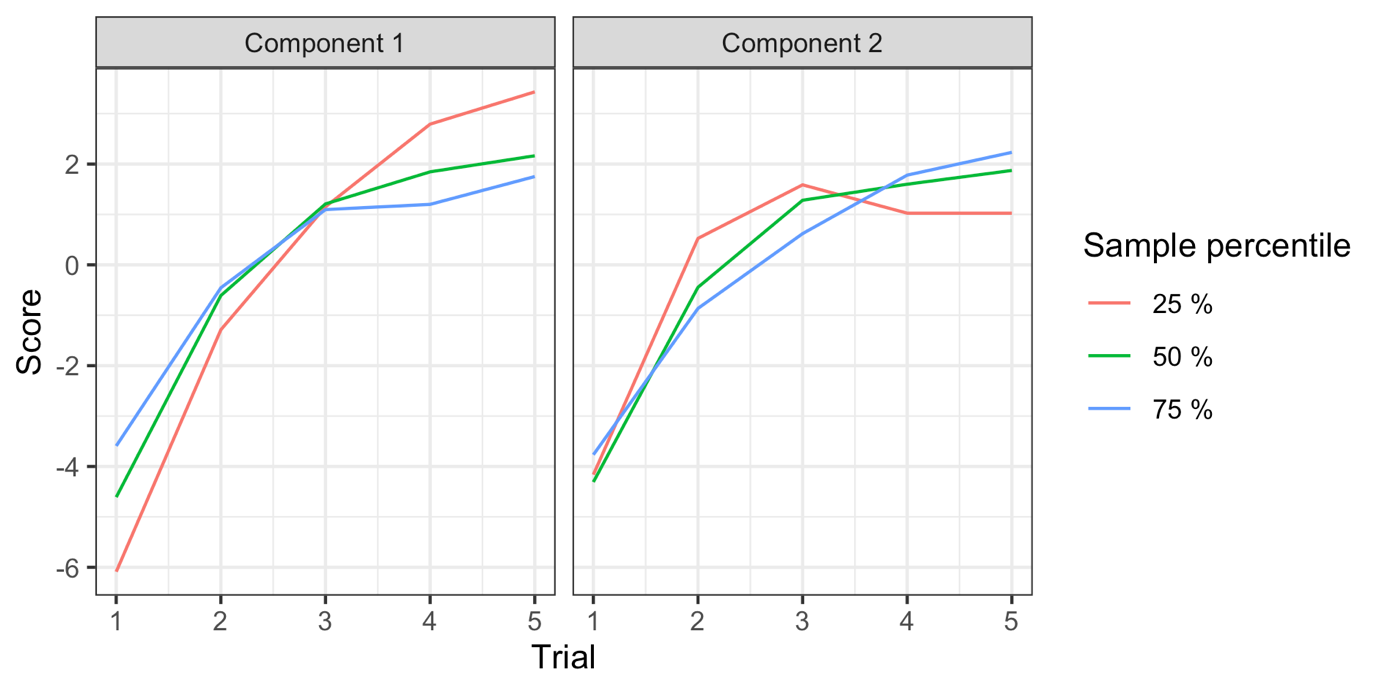


For prediction of 30-minute free recall, use of all four principal components was optimal in terms of minimizing AIC. The regression coefficients are summarized in the table below.

| Component | Estimate | 95 % CI | p-value | t-statistic |
| --- | --- | --- | --- | --- |
| 1 | -0.24 | -0.27; -0.21 | <1e-12 | -17.1 |
| 2 | 0.10 | 0.06; 0.14 | 4.8e-7 | 5.04 |
| 3 | -0.07 | -0.12; -0.02 | 0.0017 | -3.13 |
| 4 | -0.06 | -0.11; -0.01 | 0.015 | -2.44 |

In light of the principal components visualized above, the first two regression coefficients have the following interpretation.

- Component 1: Subjects with a steep learning curve (low value of component 1) have higher 30-minute free recall than subject with a less steep learning curve.
- Component 2: Subjects with a learning curve that levels off (low value of component 2) have lower 30-minute free recall than subjects whose curves have positive slopes throughout the experiment.

Although the two remaining components had significant regression coefficients, we were not able to make equally clear interpretations.

**Supplementary Figure 2 *Twin sample* phenotypic, genetic and environmental variance across the learning trials**

There is a steady increase in each variance across the trials. The increase in genetic variance is relatively greater, resulting in greater heritability, albeit alos in absolute terms greater unique environmental variance with learning.


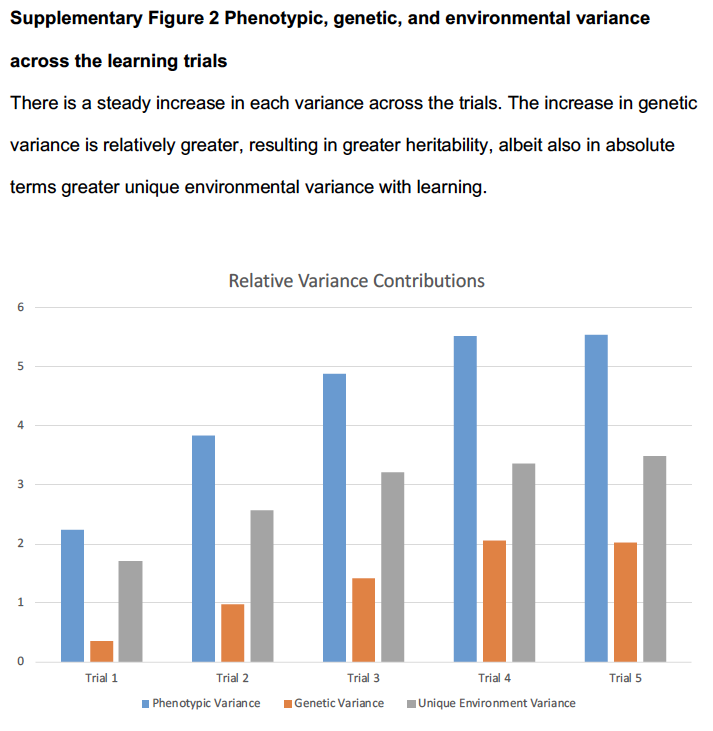


**Supplementary Methods**

**Supplementary Table 2** Distribution of number of participants from different sub-studies and scanners across time points (Tp).

|  | **Tp 1** | **Tp 2** | **Tp 3** | **Tp 4** | **Tp5** | **Tp6** |
| --- | --- | --- | --- | --- | --- | --- |
| **Sub-study** |  |  |  |  |  |  |
| MoBa | 457 | 234 | 111 | - | - | - |
| ND | 306 | 133 | 4 | - | - | - |
| CPLS | 841 | 328 | 145 | 34 | 1 | - |
| NCP | 221 | 193 | 163 | 140 | 115 | 57 |
| **Scanner** |  |  |  |  |  |  |
| Avanto 1 | 814 | 536 | 133 | 1 | - | - |
| Avanto 2 | 111 | - | - | - | - | - |
| Prisma | 136 | 56 | 105 | - | - | - |
| Skyra | 764 | 296 | 111 | 153 | 116 | 57 |

**Information on the memory training program (*Training sample*)**

The memory training program ten-week program included one in-class course session each week and eight weekly online home assignments with a minimum requirement that four be completed. The assignments involved tasks of word lists to be memorized by utilizing the Method of Loci (MoL)^1^. The method involves creating a mental travel route of a familiar place, such as one´s home. When memorizing a list of items, one visualizes placing the items along this travel route. For retrieval of the items, one imagines walking through the travel route, recollecting the items previously placed along the route. MoL has previously been shown to improve serial recall substantially in both young and older adults^2-5^. The first group session included a presentation of the project, an introduction to the MoL method with instructions, and an initial word list task consisting of 15 words. The research fellow leading the group session was available for questions and provided further explanations and repetition of instructions to ensure that all participants were able to utilize the technique. The following weekly group sessions included updating of the strategy, clarification of instructions and a word list task, which was increased by five words each week to ensure a continuous challenge. However, the participants were encouraged to individually adjust the difficulty level of the tasks both in class and of the home assignments, with the aim of achieving a challenging but manageable training level across all the participants. The home assignments were completed online and all responses in addition to time spent on the tasks were registered to a database. Both age groups underwent the same program. The number of total tasks completed was on average 48.1% in the young training group and 72.6% in the older training group.

**Supplementary References**

1 Bower, G. H. Analysis of a mnemonic device. *American Scientist* **58**, 496-519 (1970).

2 de Lange, A. G. *et al.* White matter integrity as a marker for cognitive plasticity in aging. *Neurobiology of aging* **47**, 74-82, doi:10.1016/j.neurobiolaging.2016.07.007 (2016).

3 Engvig, A. *et al.* Effects of memory training on cortical thickness in the elderly. *NeuroImage* **52**, 1667-1676, doi:10.1016/j.neuroimage.2010.05.041 (2010).

4 Kliegl, R., Smith, J. & Baltes, P. B. On the locus and process of magnification of age differences during mnemonic training. *Developmental Psychology***.26**, pp, doi:10.1037/0012-1649.26.6.894 (1990).

5 Nyberg, L. *et al.* Neural correlates of training-related memory improvement in adulthood and aging. *Proceedings of the National Academy of Sciences of the United States of America* **100**, 13728-13733, doi:10.1073/pnas.1735487100 (2003).
